# The effects of repeated intravenous iohexol administration on renal function in healthy beagles – a preliminary report

**DOI:** 10.1186/1751-0147-54-47

**Published:** 2012-08-14

**Authors:** Robert M Kirberger, Nicolette Cassel, Ann Carstens, Amelia Goddard

**Affiliations:** 1 Department of Companion Animal Clinical Studies, Faculty of Veterinary Science, University of Pretoria, Private Bag X04, Onderstepoort, 0110, Republic of South Africa

**Keywords:** Dog, Contrast induced nephrotoxicity, CIN, CT, GFR, Iohexol

## Abstract

**Background:**

Contrast induced nephrotoxicity (CIN) is a well described syndrome in humans undergoing contrast medium examinations. To date CIN has received minimal attention in the veterinary literature despite increasing use of contrast medium examinations in computed tomographic studies.

**Methods:**

This prospective study evaluated the effect of 1290 mg/kg iohexol given intravenously to 5 normal beagle dogs in a divided dose at an interval of 6–8 weeks. Renal function was evaluated by means of scintigraphically determined glomerular filtration rate (GFR) and a variety of laboratory assays.

**Results:**

Only GFR showed a significant decrease (17%) after the second injection but not to a clinically or pathologically significant level.

**Conclusions:**

No clinically significant effect of repeated contrast medium administration was determined in this limited study**.** However in dogs with reduced renal function the risk of CIN is likely to increase dramatically post contrast administration.

## Introduction

Radiographic imaging using intravascular iodinated contrast agents was the mainstay of many advanced diagnostic procedures prior to the 1990s. The advent of diagnostic ultrasound resulted in a marked decrease of abdominal and cardiac contrast procedures in many referral institutions in the ensuing years. Since the turn of the century there has been an increased use of computed tomography (CT) which is currently a popular diagnostic modality in veterinary science due to increasing accessibility, enhanced applications and its minimal invasiveness. Many of these CT studies included intravascular contrast medium administration. Computed tomography angiography (CTA), the study of vascular structures by utilizing contrast agents during the CT examination and multi-phase angiography to investigate arterial and venous phases, as well as organ perfusion have seen a marked increase in veterinary use over the last few years particularly with the advent of helical multislice CT machines. As the diagnostic advantages of these techniques are being researched [1-7] increased knowledge has lead to a dramatic increase in the use of intravascular iodinated contrast agents again, and their potential side effects must not be forgotten in routine clinical practice.

The iodinated contrast agents used in diagnostic imaging are categorized according to their physical and chemical properties [8]. First generation agents are all high-osmolar ionic monomers [8]. Their high osmolality results in increased rates of adverse reactions and they have been replaced by the safer second and third generation agents. The most commonly used second generation iodinated compounds (iopamidol and iohexol) are low osmolar, non-ionic monomers which have improved vascular tolerability and thus have fewer side effects [8]. As the radio-opacity of a contrast medium is directly related to the iodine content, the goal in developing successive generations of contrast media has been to maximize the iodine content while minimizing the osmolarity of the resulting solution. Third generation iodinated contrast media (iodixanol and iotrolan) are iso-osmolar, non-ionic dimers and are reported to be the safest contrast agents to date. However, their high cost limits their use in human patients at risk of contrast-induced nephrotoxicity (CIN) [9,10] and in particular those patients with severe renal insufficiency [11]. In contrast, other reports show no difference between second and third generation contrast agents to induce CIN [12].

Adverse reactions to iodinated intravenous contrast media have been extensively reported and investigated in humans [8,12-15]. The side effects are related to their physical and chemical properties. Side effects have been classified as non-anaphylactoid, anaphylactoid and delayed [8]. The non-anaphylactoid reactions include pulmonary edema, cardiac arrhythmias, nausea, seizures and renal failure. Anaphylactoid reactions can cause urticaria, laryngeal edema and bronchospasm. This is not a true anaphylaxis as it is not stimulated by an immunoglobulin E-mediated response and may also occur during the first exposure to the agent [8]. However, treatment is the same as for an anaphylactic reaction. The above reactions appear within 1–3 minutes whereas delayed reactions may occur up to 6 hours after exposure and include fever, pruritis, arthralgia, headache, nausea and vomiting and CIN [8].

Despite the incidence of CIN in the human population being low (<2%), regardless of the contrast medium used, [10] it is one of the leading complications of contrast use in human medicine and accounts for 10–11% of all causes of hospital acquired renal failure [14,16]. Contrast induced nephrotoxicity manifests as an abrupt decline in renal function as early as 24 hours and as long as 7–10 days following contrast administration in the absence of an alternative etiology. The patients usually remain asymptomatic. In humans, CIN is characterized by an increase in serum creatinine level of at least 44.2 μmol/L or 25% from baseline. In most cases this rise has been reported to occur 24–72 hours following exposure [8,9,12,13,16-18]. One publication cited that the creatinine levels peaked at 96 hours post contrast administration and thus CIN may be overlooked or underestimated in studies measuring creatinine concentrations at 48 hours or less [19]. Fortunately, CIN is usually self limiting with serum creatinine levels returning to near baseline values within 1–3 weeks [17]. Creatinine levels, although most commonly used, are not the ideal method of evaluating CIN as renal function must be severely compromised before elevation occurs [13]. Various risk factors for CIN, such as pre-existing renal impairment, dehydration, diabetes mellitus (independently or with associated renal disease) and advanced congestive heart failure have been identified [8,10,16,17,20,21] and thus, contrast agents may be contributory rather than causative in CIN. Co-morbidities such as hypertension, anaemia and peripheral vascular disease may also play a role [20]. Arterial injections carry a slightly higher risk of developing CIN [8] and there are conflicting reports on the effect of higher contrast agent dosages on the development of CIN [18,21]. However, in patients at risk, higher volumes are associated with higher rates of CIN [16]. A recent report concluded that human patients receiving repeat contrast CT examinations within 24 hours, had a 12.4% incidence of CIN and that an increase in the serum creatinine values between the first and second CT examination was highly associated with CIN [22].

The mechanisms of CIN have been investigated during numerous *in vitro* and *in vivo* studies. Multiple complex factors result in hypoxia of the medulla and subsequent acute tubular necrosis [16]. Three main mechanisms, namely oxidative stress, hemodynamic disturbances (vasoconstriction) and hyperosmolar effects are implicated [9,12,13]. Low oxygen tension normally exists in the renal medulla and this region of the kidney has a high metabolic rate and oxygen requirement due to active salt reabsorption in the medullary thick ascending limbs of the loops of Henle [16,20]. Contrast agents aggravate this outer medullary hypoxia as they cause enhanced metabolic activity and oxygen consumption as a result of osmotic diuresis [13]. As contrast media may be uricosuric and may increase the excretion of both oxalate and Tamm-Horsfall proteins, there is a theory that contrast nephrotoxicity may be mediated by tubular obstruction [13]. Pathological studies of animal models of CIN often reveal vacuolation of the proximal tubular epithelium [21]. Recent studies have also implicated a decrease in the antioxidant enzyme activity and/or an increase in oxygen free radicals as a putative mechanism for CIN [13]. During an *in vitro* study of the effect of contrast agents on various cell lines, the cytotoxic effect of the contrast agent causing renal cell apoptosis was identified as early as 15 minutes of incubation, reaching a maximum at 3 hours [14]. Several studies have documented a contrast induced rise in various proteins and enzymes, which although non-specific for tubular damage, do support a theory of a direct toxic effect on the tubular epithelium [21]. Studies into the pathogenesis of CIN have focused on the ischemic effects on the renal cells. It has been repeatedly demonstrated that when contrast medium is injected directly into the renal artery of both dogs and humans, there is a biphasic response in the blood flow. There is an initial very brief increase in flow, followed by a period of reduced flow lasting several minutes in normal kidneys. This reduced flow is most likely due to intrarenal vasoconstriction. Elevations of adenosine and endothelin are the main contributors to this vasoconstriction [16,20]. This vasoconstriction is then followed by an increased lipid peroxidation due to the increased production and decreased removal of free oxygen radicals, which correlates directly with a decrease in the glomerular filtration rate (GFR) [21].

In humans CIN may be limited by reducing the amount of contrast used and using fluid therapy for 3–24 hours before and 6–24 hours after the procedure [13,17,20,23]. Use of 0.9% saline at 1–3 ml/kg/hour before and 1 ml/kg/hour after the procedure is advised [20]. In some studies the addition of sodium bicarbonate appears to have had a beneficial effect [24]. The use of diuretics is generally contraindicated [17]. This creates a conundrum as diuretics are used in human excretory urography CT [25] as well as in dogs at our institution for the evaluation of ectopic ureters. These dogs may well have subclinical renal involvement and would thus be more susceptible to CIN. Vasodilating and antioxidant pharmacologic agents that have been used with varying success to limit CIN, include prostaglandins, dopamine, adenosine antagonists such as theophyline, and antioxidants, particularly N-acetylcystine, to name but a few [13,14,20,23,24,26].

There is a paucity of literature on adverse contrast agent effects in animals and in particular CIN. A recent CT textbook only mentions CIN in passing [6]. Three decades ago, a study was done to determine the effect of three increasing dosages excretory urograms at 48 hour intervals on GFR in dogs as determined by blood urea nitrogen (serum urea), serum creatinine and endogenous creatinine clearance [27]. The contrast agent used was sodium iothalamate, a first generation contrast agent. Only the endogenous creatinine clearance decreased in 50% of the dogs, indicating some decrease in GFR following contrast urography. Acute renal failure is described in a 14-month-old dog 190 hours after excretory urography with a first generation contrast agent. The dog recovered after four days of intensive treatment [28]. Only one experimental study to date has looked at CIN in control and gentamycin induced renal dysfunction in dogs and the effect of a low dose of dopamine and 0.9% saline to reduce potential CIN and improve excretory urography image quality [29]. Serum chemistry assays included serum urea and creatinine. This study showed increased serum urea levels 72 hours after injection of contrast medium only in dogs with normal renal function. Interestingly dogs with impaired renal function showed no significant change in serum urea values.

More work has been done on anaphylactoid reactions. A severe reaction in 2 anesthetized dogs undergoing CT angiography with first generation ionic intravenous contrast media has been described [30]. One dog developed hypertension, bradycardia, apparent bronchospasm and diarrhea and the other became hypotensive, tachycardic and had ventral abdominal and pelvic limb erythema and diarrhea. Both dogs recovered after intensive treatment. Recent retrospective studies reported on the effect of first and second generation contrast media CT studies in dogs and cats on heart rate, peak systolic blood pressure and serum urea and creatinine concentrations [31,32]. There were minor hemodynamic changes with some serum biochemistry changes which were difficult to interpret due to low numbers and varying histories and sampling intervals. The serum urea and creatinine post injection time measurements were often weeks to months later and thus were of no value to determine possible CIN within 24–96 hours post injection. One equine report using intravenous contrast medium in an 18-year-old Dutch warm blood could be found. The horse received a total dose of 480 ml of first generation contrast agent for a head CT. The horse developed a severe anaphylactoid reaction (bronchospasm, hypoxemia, hypotension and sweating), but recovered after intensive monitoring and treatment [33]. More recently a report on the effects of distal extremity limited volume intra-arterial contrast medium administration in horses showed 4% developing anaphylactoid reactions (urticaria and edema) whilst 5% developed a temporary increase in heart rate and blood pressure [34].

Glomerular filtration rate is a good measure of renal function as it is directly proportional to the number of functioning nephrons [31]. It is considered to be the best parameter to estimate renal function for diagnosis and prognosis in clinical situations [35]. Glomerular filtration rate cannot be measured directly but is estimated using the uptake or clearance of a filtration marker. Filtration markers for GFR must satisfy several physiologic requirements which include: 1) being freely filtered by the glomeruli, 2) not being bound to plasma protein and 3) not being reabsorbed, secreted or metabolized by the renal tubules [36-38]. Many methods have been developed and described, including endogenous and exogenous creatinine clearance, C-inulin clearance, iodinated contrast medium clearance, contrast enhanced renal CT and ^99m^Tc-diethylenetriamine pentacetic acid (^99m^Tc-DTPA) scintigraphy [36,37,39]. The traditional gold standard for measuring GFR is the determination of exogenous inulin clearance; however this method is technically difficult as it requires timed collection of both urine and plasma [36,38]. The scintigraphic GFR technique is quick and non-invasive, does not require blood or urine samples and can also asses individual kidney GFR [36,39,40]. However it requires expensive equipment, an authorized laboratory and technical expertise. Although global and regional GFR can be measured using contrast enhanced CT, the results in a canine study revealed lower CT GFR values when compared to scintigraphy [37]. It was hypothesized that the difference may have been as result of the anesthetic effects during the CT on blood pressure, cardiac output and renal vascular resistance as well as potential nephrotoxic effects of the iohexol.

The scintigraphic method can be more variable than the plasma clearance methods, due to the short imaging time, environmental factors affecting GFR and inter-operator variability [35,36,40-42]. The dynamic renal scintigram is based on 2 minute sampling time during which auto regulatory mechanisms may alter renal blood flow and pressure [36]. Day-to-day variability’s include gamma camera variations, stress (dogs should be acclimatized to the scintigraphic procedure), hydration status, water and food intake and composition, particularly protein content. The amount of protein binding of the radiopharmaceutical is also an important factor to consider as the radiopharmaceuticals bound to protein is not available for GFR and will result in a false lower GFR value. The amount of protein binding of ^99m^Tc-DTPA is said to be low in dogs and cats (10–15%) and does not significantly influence the GFR [36,40]. Interobserver variability of GFR can be overcome by using semi-automated methods to determine the region of interest (ROI) and by averaging three measurements made by one observer [35]. All of the above result in some degree of variation of results in normal kidneys. In animals with compromised renal function the values will be more consistent as the kidneys are operating at or near their maximum GFR [36]. Normal values in the dog are generally above 3 ml/min/kg and vary from 3.13 ± 0.53 to 3.97 ± 0.72 and 4.05 ± 1.1 ml/min/kg [36,37,41,43] Subclinical renal insufficiency has GFR values between 1.2 and 2.5 ml/min/kg. Animals with levels below 1–1.3 ml/min/kg often have elevated serum urea and creatinine concentrations [36].

Various serum chemistry assay and urinalysis findings are evaluated when a suspicion of acute renal damage exists. Serum creatinine and serum urea concentrations provide an estimation of GFR but have poor sensitivity in that they do not become elevated until there is significant renal insufficiency [36,38,39]. Azotemia may also be influenced by extraneous factors. Serum inorganic phosphorus (SIP) can be maintained within relatively normal limits for some time by the endocrine responses involved in renal secondary hyperparathyroidism. Similar to serum creatinine and serum urea concentrations, SIP shows poor sensitivity as it is thought that approximately 85% of the GFR must be lost before persistent hyperphosphatemia develops [44]. Urinalysis findings, such as glucosuria and proteinuria, can also alert one to renal damage. In various studies in dogs with gentamycin induced nephrotoxicity, 24 hour urinary gamma-glutamyl transpeptidase (GGT) and spot urine sample GGT:creatinine ratio were found to be more sensitive and reliable methods of detecting tubular damage, particularly in the early phase, before other serum and urine findings where altered [45,46]. Urine protein:creatinine (UPC) ratio is useful to quantify urinary protein loss from renal origin [47]. However in dogs with stable proteinuria UPC values in the lower ranges may differ as much as 40% limiting its value in transient nephropathy [47].

The objective of this study was to determine the effect on renal function of two standard dosages of intravenous iohexol 6–8 weeks apart in normal dogs by using scintigraphically determined GFR and a variety of laboratory assays. The study ran concurrently with a CTA study that will be reported elsewhere. The hypotheses posed were: GFR will decrease, serum urea, creatinine, and SIP concentrations as well as urine GGT:creatinine and UPC ratios will increase after the two CTA procedures. The authors acknowledge that 5 dogs are a small sample population and that the expected minor changes in renal function induced by the contrast agent would require a larger number of dogs to give the study the required statistical power in some of the tests. However, as a trial was already being undertaken by the authors to compare different CTA techniques in 5 dogs, it provided an ideal opportunity to do a preliminary prospective investigation on the effects of contrast media on renal function, and in particular GFR, which is much more sensitive than routine laboratory evaluations of renal function.

The protocol was approved by the University of Pretoria animal use and care committee as well as research committee (Protocol number V044-09).

## Materials

### Subject material

Five normal adult purpose bred laboratory Beagles, three males and two females, all but one male and one female sterilized, were used. Dogs had a mean age of 48 ± 15 months (range 37–68) and weight of 13.81 ± 2.4 kg (range 10.4–16.1). The dogs were determined to be healthy 4 days before the onset of the trial based on normal clinical examination, thoracic radiographs, abdominal ultrasound, blood pressure (non-invasive oscillometric technique) and a complete blood count. Renal function normality was assessed as reported below. The dogs were housed in the Onderstepoort Veterinary Academic Hospital during each of the 7 day study periods and were fed a commercial diet twice daily and given water *ad libitum*. The animals were fasted for 12 hours preceding the GFR and CTA studies and water was provided *ad libitum* up to sedation. In between the study periods the dogs were housed at the University of Pretoria Biomedical Research Centre or the Onderstepoort Teaching Academic Unit.

### Clinical pathology

Routine urine analysis as well urine GGT, protein and creatinine values were determined. From the latter GGT:creatinine and UPC ratios were calculated. A specific serum biochemistry panel (serum urea, creatinine, SIP, sodium and potassium) was performed on all the dogs as part of the initial screening, 72 hours prior to, 72 hours and 2 weeks after each CTA. Albumin and globulin concentrations were obtained as part of the initial screening and again 72 hours before and 72 hours after the second contrast medium injection.

### Iohexol administration

All dogs were part of a separate thoracic CTA trial comparing bolus tracking and test bolus techniques. Each dog received each CT technique 6–8 weeks apart starting with bolus tracking with each dog receiving 2 ml/kg iohexol (300 mgI/ml – total dose 600 mg/kg) (Omnipaque, Adcock Ingram Healthcare, Bryanston South Africa) administered with a pressure injector at 3 ml/sec. Test bolus dogs received a manually injected test bolus of iohexol 300 mgI/ml (15% or 0.3 ml/kg of the total dose of 2 ml/kg) followed by an additional dose of 2 ml/kg administered with a pressure injector at 3 ml/sec. All injections, including the test bolus, were followed by a manual saline chaser of 1 ml/kg. The total dose received for the test bolus technique was thus 690 mg/kg and the combined trial dose for each dog was 1290 mg/kg.

### Scintigraphic GFR

The scintigraphic GFR was performed on all the dogs 72 hours prior to, and 72 hours after each CTA.

The anesthetic protocol was adapted from previous studies to minimize the effects of drugs on the GFR [42,43]. Dogs were premedicated with intravenous butorphanol (Torbugesic®, Pfizer laboratories, Sandton, South Africa) at 0.4 mg/kg and diazepam (Aspen Pharmacare, Woodmead, South Africa) at 0.2 mg/kg. This combination has minimal effect on blood pressure so as to reduce the risk of aggravating a potential CIN. Induction followed 5 minutes later with intravenous propofol (Fresenius Kabi, Midrand, South Africa) to an effective level of deep sedation. Additional doses of propofol were given if required to keep the dog motionless. Propofol was added to the regimen as it has been shown that butorphanol and valium on their own may not result in sufficient sedation in young healthy dogs [43]. A gamma camera (Equine scanner HR, Medical Imaging Electronics, Burkhard Rauchfub. Haupt str. 112. 23845 Seth, Germany) fitted with a Low Energy All Purpose (LEAP) collimator and a 64 by 64 x 16 matrix was used.

The GFR studies were done in a similar manner to previously described techniques [35,38,41], and at the same time of the day within a 2 hour window between 10 and 12 pm. Commercially supplied ^99m^Tc-DTPA (Syncor 917 Morkels close, Unit F81, Allandale Park, Midrand, South Africa) was administered as a 1 ml bolus with the dose calculated by subtracting the dose remaining in the syringe after injection from the full syringe dose. As all counts were made within 10 minutes of injection, radioactive decay measurements were deemed unnecessary [39,48]. Mean dose administered was 142 ± 21.4 MBq (range 97.2–172.1). The patient was positioned in left lateral recumbency with the gamma camera positioned dorsal to the kidneys. The ^99m^Tc-DTPA was manually injected into the cephalic vein followed by a 1 ml/kg saline flush. Image acquisition was initiated simultaneously with injection. Images were acquired at 6 second intervals for 3 minutes. After the dynamic acquisition the camera was rotated 90 degrees and a 30 second right lateral static image was made for renal depth correction calculations. Images were displayed sequentially and corrected for motion when necessary using motion correction software. Composite images of the dynamic series were used to form a single image of the kidneys with sufficient counts to define the renal edges. A manual renal ROI was drawn allowing up to 1–2 pixels peripheral to the high intensity uptake to be included. Background (1–2 pixels cranial and caudal to each kidney) rectangular 3 × 7 pixel regions of interest were drawn avoiding other isotope uptake areas. A time activity curve was created by applying the regions of interest to the dynamic data from 1 to 3 minutes to avoid pelvic isotope uptake hampering interpretation after 3 minutes [39,48]. Depth correction was used to determine individual renal function to the total renal function by the computer. To reduce observer variability all measurements were made by one investigator (NC) and repeated three times and a mean value obtained. All dogs were kept in isolation for 24 hours following the scintigraphy.

### Data and statistical analysis

Data were captured on a spreadsheet and statistically analyzed using the SPSS 17.0 statistics package (IBM Southern & Central Africa, Private Bag x9907, Sandton, South Africa). Results were expressed as mean +/− standard deviation (SD) and range. Statistical tests were performed between the 6 times the dogs were evaluated for all the tests except for the GFR which only had 4 test times. Because of the low number of dogs tested, non-parametric tests were run, namely Friedman’s ANOVA and if there was a significant difference Wilcoxon’s signed rank test were performed on these data to determine whether there was significant difference between the means at different testing times. The significance level for all tests was set as P < 0.05. Power analysis done for the GFR, which had very low standard deviations revealed the number of dogs tested to be sufficient.

## Results

### Clinical pathological values

Albumin, globulin, sodium and potassium values remained within normal limits throughout the study.

**Figure 1:**
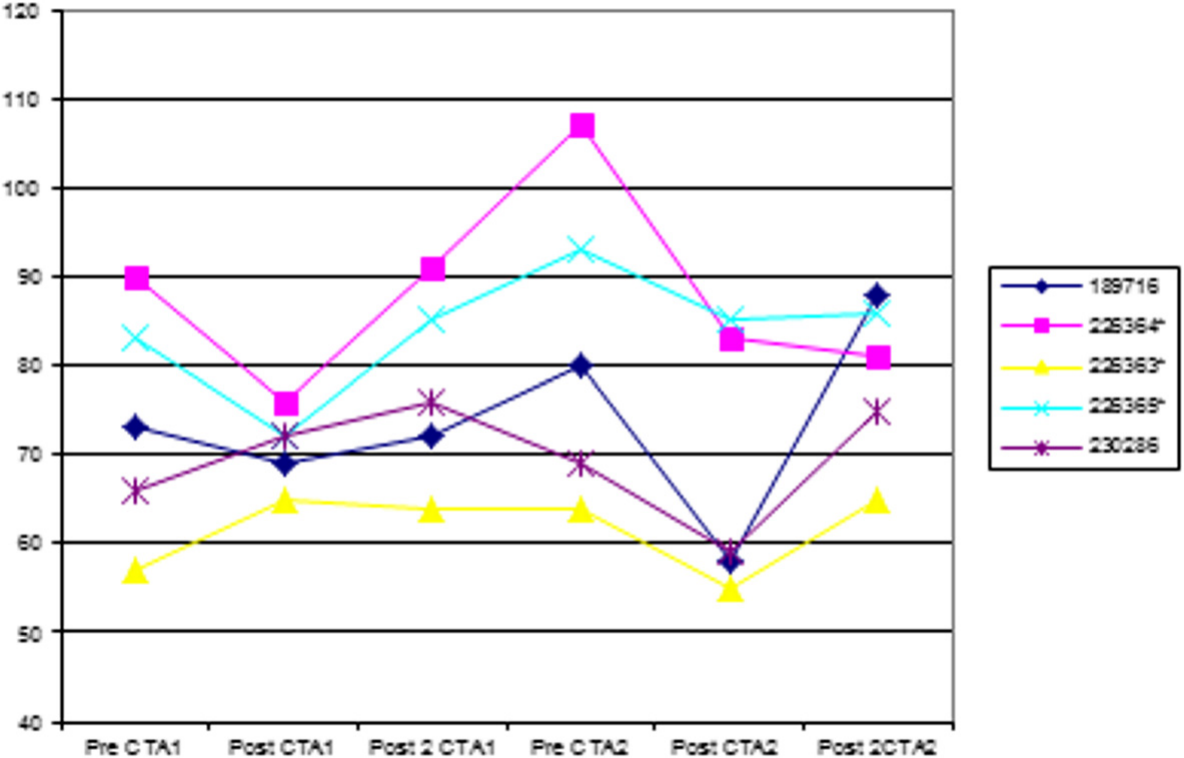
**Graph showing serum creatinine values in μmol/L pre- and post injection of contrast medium 6–8 weeks apart.** See text for time intervals. Reference values for our laboratory are 40–133 mmol/L.

Serum creatinine concentration remained within the normal limits of 40–133 μmol/L with a range of 55–107 μmol/L. There was no significant difference among the combined data (P = 0.053). There was a significant increase of serum creatinine concentration values from 72 hours before the first CTA to 72 hours before the second CTA (73.8 *vs* 82.6 μmol/L, respectively; P = 0.031), and a significant decrease between the 72 hours before the second CTA and 72 hours after the second CTA (82.6 *vs* 68 μmol/L, respectively; P = 0.031) (Figure 1).

The serum urea values remained within the normal limits of 3.6–8.90 mmol/L except for one dog with a value of 9.4 mmol/L, 72 hours after the second CTA. One pre first CTA sample could not be evaluated for technical reasons. The highest mean value for the test periods was 6.36 mmol/L 72 hours before the second CTA compared to an initial value of 5.7 mmol/L. There was no statistically significant difference between the various groups’ combined data (P = 0.742).

**Figure 2:**
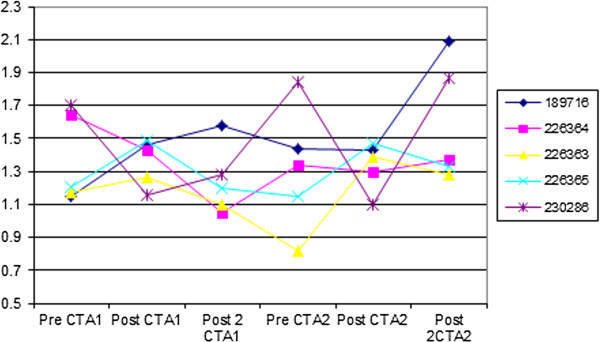
**Graph showing serum inorganic phosphate values in mmol/L pre- and post injection of contrast medium 6–8 weeks apart.** See text for time intervals. Reference values for our laboratory are 0.90–1.60 mmol/L.

The SIP values remained within normal limits of 0.90–1.60 mmol/L except for 4 random values of which 3 were above and 1 below normal. There was no statistically significant difference between the various groups’ combined data (P = 0.195), but there was a significant increase between the 2 weeks post first CTA and 2 weeks post second CTA (1.24 *vs* 1.59 mmol/L, respectively; P = 0.031), and between the 72 hours pre second CTA and 2 weeks post second CTA (1.38 *vs* 1.59 mmol/l, respectively; P = 0.031) (Figure 2).

Urine creatinine concentrations were not determined in 2 pre first CTA samples due to technical reasons. The urinary GGT:creatinine remained fairly constant with a mean of 0.20 for the whole study and a minimum mean of 0.18 and maximum mean 0.23 during each test period. All these values were well below the reported normal value of 0.39 ± 0.18 [46]. There was no statistically significant difference between the various groups’ combined data (P = 0.198). The UPC ratios also remained well within normal limits of <0.5 [49] with the mean value for each test period being 0.11 (range 0.08-0.13). There was a trend for the values to increase 2 weeks after the first injection and 3 days after the second injection, but this was not significant.

**Figure 3:**
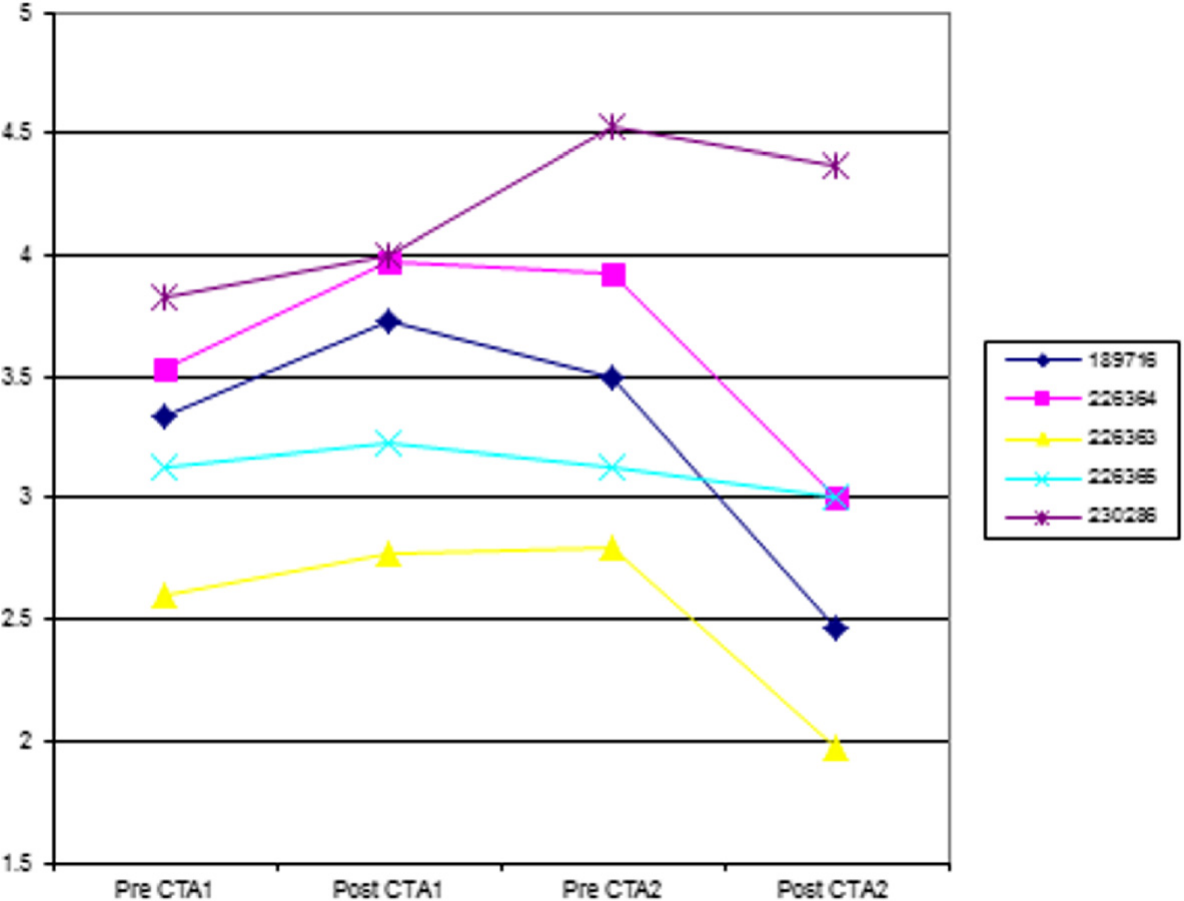
**Graph showing GFR values in ml/min/kg 72 hours pre- and post injection of contrast medium 6–8 weeks apart.** Normal reported values are greater than 3.13 ± 0.53 ml/min/Kg.

The ^99m^Tc-DTPA GFR scintigraphy showed a significant difference between the combined groups’ values (P = 0.016), with the mean GFR value 72 hours prior to first CTA of 3.284 increasing significantly to 3.54 ml/min/kg 72 hours after the first CTA procedure (P = 0.031) and then dropping significantly from 72 hours before the second CTA from 3.578 to 2.962 ml/min/kg 72 hours after the second CTA (P = 0.031) (Figure 3).

## Discussion

There was no significant difference between the various groups’ combined data for the urine and serum biochemistry panel changes and the only significant changes between individual data groups over the period of the trial were serum creatinine and SIP concentrations. The 3 samples that could not be evaluated for technical reasons were all pre-first CTA procedure and hence could not really affect the outcome of the rest of the sample analyses. The UPC ratio was the only urine test that showed an increasing trend. Serum creatinine concentrations showed a significant increase between 72 hours before the first CTA and 72 hours before the second CTA which was an interval of 6–8 weeks with no significant changes 72 hours after each injection as expected. The described increase was still within normal limits and was an increase of 8.8 μmol/L (12%). Compared to human studies this increase is not clinically significant as CIN is characterized by an increase in serum creatinine concentration of at least 44.2 μmol/L or 25% from baseline [12,17,18]. An additional confounder was the significant decrease in concentrations between 72 hours before and 72 hours after the second contrast injection (82.6 *vs.* 68 μmol/L, respectively) when most human studies show an increase 24–72 hours following exposure [8,9,12,13,16-18]. Creatinine concentrations have been reported to peak up to 96 hours post contrast administration and a possible elevation may theoretically have been missed [19]. Fortunately CIN is usually self limiting and if an elevation occurred this usually returns to near baseline values within 1–3 weeks [17] making the 6 weeks later elevation also unlikely to be clinically significant.

The serum urea concentrations showed no significant increase confirming the insensitivity of this test to detect early renal dysfunction [35,38,39].

The SIP concentrations also showed some significant increases but not beyond normal levels and not in the expected time intervals of 72 hours post injection, again making these findings unlikely to be clinically significant.

The GFR increased by 0.26 ml/kg/hour from 3.284 to 3.54 ml/kg/hour, 72 hours after the first injection. This increase was unexpected and was statistically significant but was unlikely to be clinically significant as GFR variations may be due to several causes. These include day-to-day variability and technical errors [41] or can be attributed to renal physiologic homeostatic adjustments [50]. Additionally, scintigraphically determined GFR is based on a 2 minute sample and GFR can vary considerably over short time intervals due to autoregulation [50]. The only possible clinically significant value of the whole trial was the decrease of GFR by 0.6 ml/kg/hour from 3.56 to 2.96 ml/kg/hour from 72 hours before to 72 hours after the second injection. This is below the normal values quoted in most studies [36,37,41] but within one standard deviation of the lowest reported normal value [43].

However the decrease in GFR should alert clinicians to the potential of CIN. It is suggested that all patients undergoing CT angiography should at least have their serum urea and creatinine concentrations, and if available UPC ratios, checked prior to the procedure. Based on human studies, dogs with elevated values believed to be of renal origin as well as diabetic dogs are likely to be at risk. These patients should be placed on an intravenous Ringer’s infusion at 1–3 ml/kg/hour for at least 3–6 hours before the angiography and 1 ml/kg/hour for 3 hours afterwards. Normal dogs should also be placed on maintenance fluids for at least 2 hours before and after the procedure. Additionally, epinephrine and intravenous prednisolone sodium succinate must be on hand to treat anaphylactoid reactions.

The effect of propofol on cardiorespiratory parameters is contradictory in the literature. One report commented that no clinically significant changes in blood pressures or heart rates were observed when propofol was used in combination with diazepam [51]. In another study investigating the anesthetic cardiopulmonary effects of propofol in dogs premedicated with butorphanol, atropine and medetomidine, a transient decrease in blood pressure was noted after propofol injection [52]. However in sedation related systemic hypotension GFR is usually maintained at normal levels by maintenance of renal blood flow [43]. So although the effects of the balanced anesthesia used in the study would require further investigation to further define the effects on blood pressure and subsequently GFR, each dog served as its own control and the same anesthetic protocol was used during each scintigraphic examination. Thus a trend in the GFR would still be identified. The only concern regarding any potential effects on the use of propofol would be in a patient with subclinical renal insufficiency as a decrease in blood pressure may exacerbate the condition.

The limitations of this study are the small number of dogs with only 4 GFR studies done on each dog. However, even when power analyses were conducted on the GFR results of the 5 dogs, the results were such that the number of dogs were deemed to be sufficient. It may have been that CIN was present after injection of contrast medium but that it occurred later than the 72 hour GFR determination but additional 96 hours post injection GFR determinations was also not within the context of the overall study. However the study did show that 2 doses of iohexol given 6–8 weeks apart did not have a major influence on renal function in these 5 normal dogs. The study was done on normal dogs and the effects of contrast medium in dogs with impaired renal function can only be speculated upon based on human studies. To perform a similar GFR study in clinical case material would be logistically challenging and to create renal damage for an experimental study would be ethically questionable.

## Conclusion

The null hypotheses of increased serum urea, creatinine and SIP concentrations and increased urine GGT: creatinine and UPC ratios after the two CTA procedures could not be proven in this limited study. A 17% decrease in GFR was shown after the second contrast medium injection. Whether this was a clinically significant induction of CIN is difficult to confirm/conclude taking into consideration the potential variations in GFR. No GFR studies could be found in the human literature to detect changes in renal function in CIN cases and all human research is based on elevated creatinine values which are known to only increase when significant renal change has occurred. The final mean GFR of 2.96 ml/kg/hour in this study is still not believed to be clinically significant as subclinical renal insufficiency has reported GFR values between 1.2 and 2.5 ml/min/kg [36]. However the potential of CIN should be considered in all patients undergoing contrast medium studies, particularly if reduced renal function is present, and appropriate preventative measures instituted.

## Abbreviations

Tc-DTPA: ^99m^Tc-diethylenetriamine pentacetic acid; CIN: Contrast induced nephrotoxicity; CT: Computed tomography; GFR: Glomerular filtration rate; GGT: Gamma-glutamyl transpeptidase; ROI: Region of interest; UPC: Urine protein:creatinine.

## Competing interests

The authors declare they have no competing interests.

## Authors’ contributions

RK con;tributed to conception and design of study and wrote manuscript, NC acquired all data and contributed to design of study and manuscript, AC did the statistical analysis and contributed to the design of the study and AG contributed to design of study and interpretation of laboratory results. All authors have read and approved the manuscript.

## Authors’ information

RK: BVSc, MMedVet(Rad), DipECVDI, Head of Diagnostic Imaging Section.

NC: BVSc, MMedVet(Diag Im).

AC: BVSc, MMedVet(Chirg), MMedVet(Diag Im), DipECVDI.

AG: BVSc, MMedVet(Clin Lab Diag), Head of Clinical Pathology Section.
